# SAXS/WAXS data of conformationally flexible ribose binding protein

**DOI:** 10.1016/j.dib.2023.109932

**Published:** 2023-12-09

**Authors:** Jagrity Choudhury, Kento Yonezawa, Anu Anu, Nobutaka Shimizu, Barnali Chaudhuri

**Affiliations:** a GN Ramachandran Protein Center, CSIR Institute of Microbial Technology, Chandigarh 160036, India; b Academy of Scientific and Innovative Research (AcSIR), Anusandhan Bhawan, 2 Rafi Marg, New Delhi 110001, India; c Structural Biology Research Center, Institute of Materials Structure Science, High Energy Accelerator Research Organization (KEK), 1-1 Oho, Tsukuba, Ibaraki 305-0801, Japan; d Photon Factory, Institute of Materials Structure Science, High Energy Accelerator Research Organization (KEK), 1-1 Oho, Tsukuba, Ibaraki 305-0801, Japan

**Keywords:** Small-angle X-ray scattering, Wide-angle X-ray scattering, Ribose binding protein, Conformational flexibility

## Abstract

Modern artificial intelligence-based protein structure prediction methods, such as Alphafold2, can predict structures of folded proteins with reasonable accuracy. However, Alphafold2 provides a static view of a protein, which does not show the conformational variability of the protein, domain movement in a multi-domain protein, or ligand-induced conformational changes it might undergo in solution. Small-angle X-ay scattering (SAXS) and wide-angle X-ray scattering (WAXS) are solution techniques that can aid in integrative modeling of conformationally flexible proteins, or in validating their predicted ensemble structures. While SAXS is sensitive to global structural features, WAXS can expand the scope of structural modeling by including information about local structural changes. We present SAXS and WAXS datasets obtained from conformationally flexible d-ribose binding protein (RBP) from *Escherichia coli* in the ribose bound and unbound forms. SAXS/WAXS datasets of RBP provided here may aid in method development efforts for more accurate prediction of structural ensembles of conformationally flexible proteins, and their conformational changes.

Specifications Table

**Table:** 

Subject	Structural Biology
Specific subject area	Small-angle X-ray scattering, Wide-angle X-ray scattering
Data format	Raw, Analyzed
Type of data	Buffer subtracted SAXS and WAXS data in .dat format
Data collection	Purified proteins (with or without added ribose sugar) were snap-frozen in liquid nitrogen and shipped in a dry-shipper to the experimental stations. SAXS data (at 3 different protein concentrations) and WAXS data were collected for RBP and RBP-ribose complex. SAXS data was collected at beamline BM29, European Synchrotron Research Facility, Grenoble, France. WAXS data was collected at beamline BL-15A2, Photon Factory, High Energy Accelerator Research Organization, Tsukuba, Japan.
Data source location	Council of Scientific and Industrial Research – Institute of Microbial Technology (CSIR-IMTECH), Sector-39a, Chandigarh, India
Data accessibility	Repository name: Small Angle Scattering Biological Data Bank (SASBDB) [1] Data identification number: SASDTB2, SASDTC2, SASDTD2, SASDSB9, SASDSC9, SASDSD9, SASDSE9, SASDSF9, SASDSG9 Direct URL to data: https://www.sasbdb.org/data/SASDTB2/50y7zrcal2 https://www.sasbdb.org/data/SASDTC2/ib78rkwuol https://www.sasbdb.org/data/SASDTD2/ptra1gqphh https://www.sasbdb.org/data/SASDSB9/rlyjmkw3v9 https://www.sasbdb.org/data/SASDSC9/n25j3idnj1 https://www.sasbdb.org/data/SASDSD9/gc9wf77u23 https://www.sasbdb.org/data/SASDSE9/szubkyqqwk https://www.sasbdb.org/data/SASDSF9/avaiglsbg4 https://www.sasbdb.org/data/SASDSG9/pnlv223tuo

## 1 Value of the Data

• The present SAXS/WAXS data can be used for validating the results of the current efforts to use Alphafold2 to predict conformational variants [2,3].
• The present SAXS/WAXS data, together with available crystal structures of RBP [4], [5], [6], [7] can be used for SAXS/WAXS supported method development efforts [8], [9], [10], [11], [12], [13].
• Since very few WAXS data sets are available in the public data repositories, the present datasets can aid in method development for accurate calculation of WAXS data from co-ordinates of the structures [14].

## 2 Data Description

**Fig. 1:**
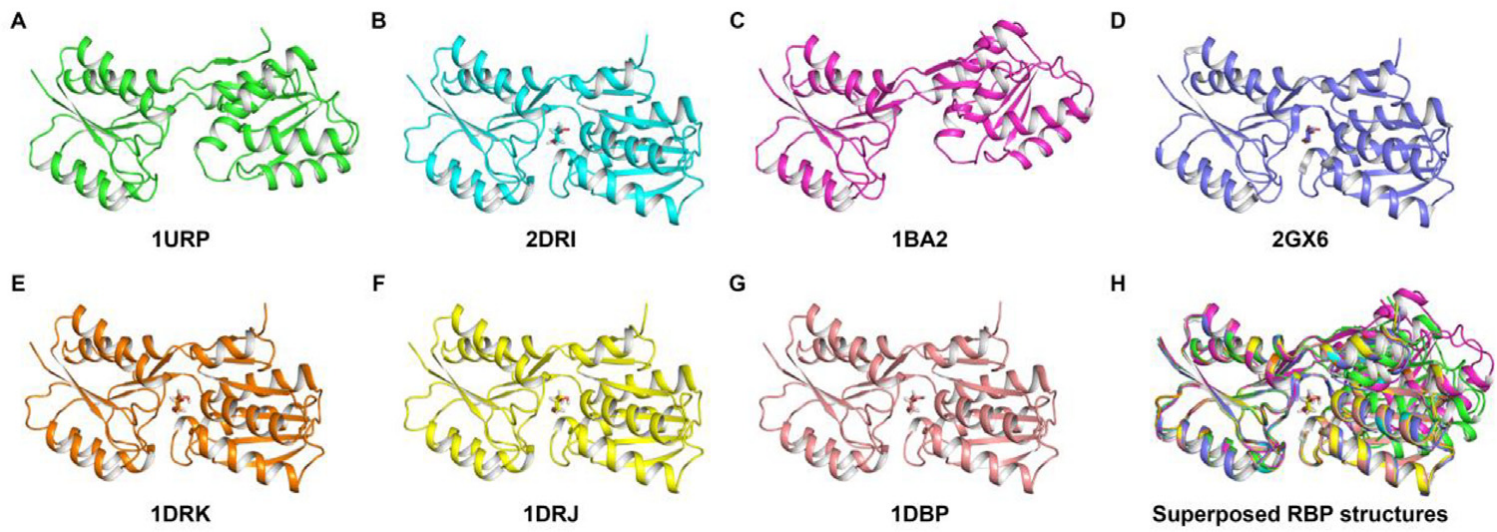
Crystal structures of RBP, shown in cartoon representation. (A) PDB ID: 1URP (Green), (B) PDB ID: 2DRI (Cyan), (C) PDB ID: 1BA2 (D67R mutant, Magenta), (D) PDB ID: 2GX6 (Purple), (E) PDB ID: 1DRK (I132T/G134R mutant, Orange), (F) PDB ID: 1DRJ (G134R mutant, Yellow), (G) PDB ID: 1DBP (G72D mutant, Pink). (H) Superposed structures of RBP (structures were aligned to the N-terminal domain of 1URP using residues 26–126, C_α_ atoms only, RMSD 0.24–0.43 Å). PyMOL (The PyMOL Molecular Graphics System, Schr⍤dinger) was used for structure superposition. Bound ribose is shown as a stick in B, D, E, F, G and H and is colored according to the elements with oxygen as red and carbon as the color of the backbone chain.

RBP, which is the periplasmic ribose-binding component of bacterial, ribose-specific ABC transporter, is a two-domain, flexible protein with a hinge adjacent to the sugar binding cleft at the domain interface [4], [5], [6], [7]. A number of crystal structures of RBP have been determined previously in both sugar-bound and free forms [4], [5], [6], [7] (Fig. 1). Since RBP is well-characterized, SAXS and WAXS datasets of RBP in free and ribose-bound forms provide a valuable dataset for method developers.

**Fig. 2:**
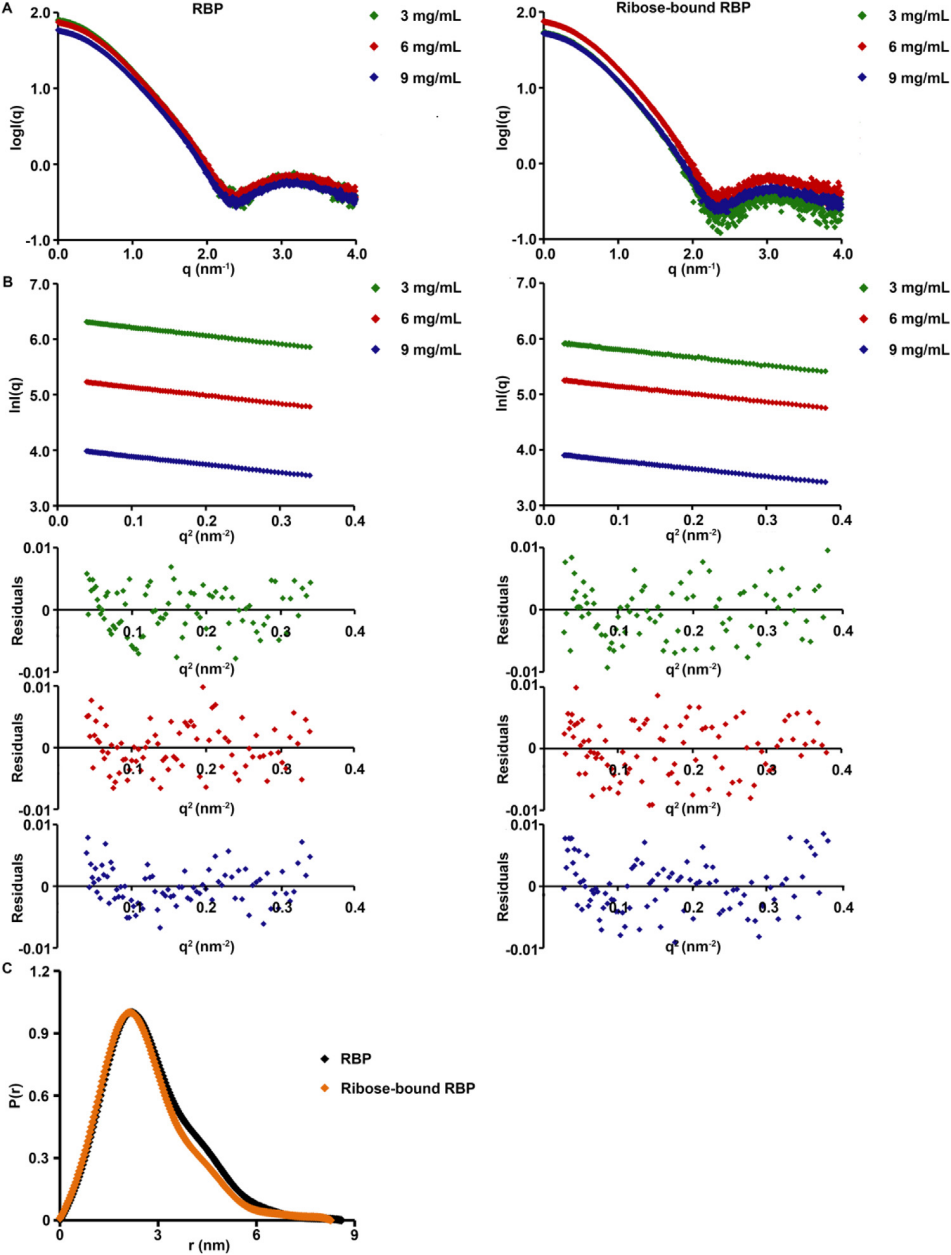
SAXS data of RBP (left panel) and ribose-bound RBP (right panel). (A) LogI(q) *versus* q plots (I is intensity in arbitrary unit and q is momentum transfer in nm^−1^, *q* = 4πsin(θ)/λ, where λ is the X-ray wavelength and 2θ is the scattering angle) at three different protein concentrations. (B) Guinier plots (lnI(q) *versus* q^2^) and corresponding residual plots of the SAXS data shown in A. The Guinier plots are shifted on the y-axis for better visualization. (C) Pair distribution function (P(r)) *versus* r (pair-wise distance in nm) plot for RBP with and without ribose, calculated using following boundary conditions: P(r) = 0 at *r* = 0 and at *r* ≥ D_max_, where D_max_ is the estimated maximum particle diameter (Table 2). The pair distribution function plot is normalized to a maximum value of 1 in the y-axis.

**Fig. 3:**
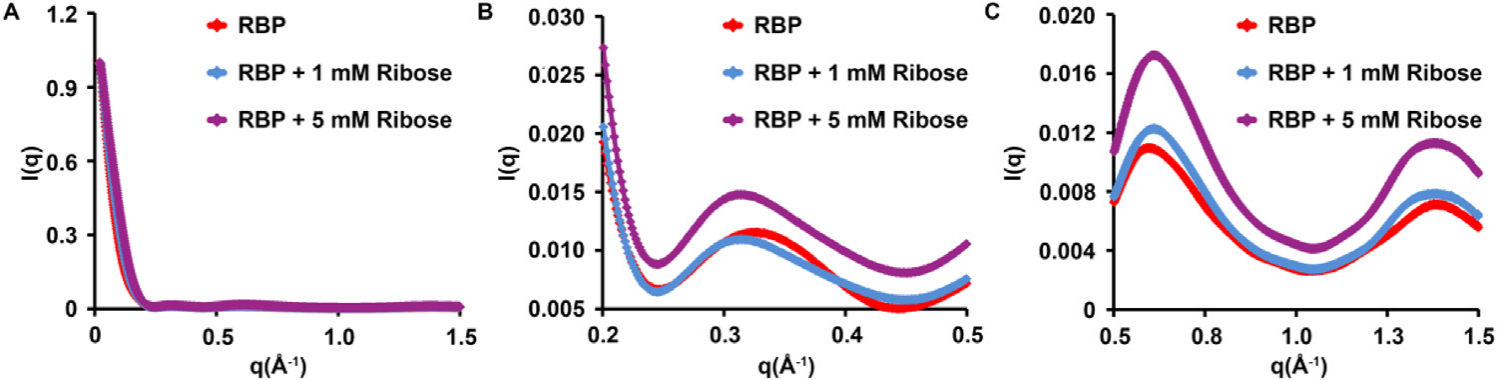
WAXS data of RBP and ribose-bound RBP. X-ray scattering intensity I in various q-ranges are shown. All the intensity data are normalized to a maximum value of 1.0. (A) q range of 0.02 *Å*^−1^ to 1.5 *Å*^−1^. (B) q range of 0.2 *Å*^−1^ to 0.5 *Å*^−1^. (C) q range of 0.5 *Å*^−1^ to 1.5 *Å*^−1^.

SAXS data were collected for both ligand bound and unbound RBP at three different protein concentrations (Fig. 2A; Table 1). Guinier analysis was performed to estimate the radius of gyration (R_g_) and evaluate data quality (Fig. 2B; Table 2). Average R_g_ for RBP was 2.10 ± 0.01 nm. In the presence of ribose, the average R_g_ for RBP was 2.05 ± 0.02 nm. RBP has been characterized by SAXS before, showing a similar, small change in R_g_ between the sugar-bound and sugar-free states [15]. Maximum particle diameters (D_max_) were estimated from the pair distribution function. A modest difference in the average D_max_ of RBP (8.43 ± 0.15 nm) and that of RBP bound to ribose (7.76 ± 0.47 nm) was observed (Fig. 2C; Table 2). Comparison of the WAXS data obtained from RBP and RBP-ribose showed expected difference consistent with conformational change between the two sets (Fig. 3).

**Table 1:** Experimental details for (a) SAXS and (b) WAXS experiments. The table contains the detailed parameters used for data collection and data processing.

a) Experimental parameters for SAXS			
Protein	RBP	RBP + 1 mM ribose	
Experimental station	BM29, ESRF		
Detector distance (m)	2.87		
Exposure time (s)	1 (10 acquisitions)		
Wavelength (Å)	1		
Background buffer	50 mM Tris pH 7, 50 mM NaCl, 10% glycerol	50 mM Tris pH 7, 50 mM NaCl,10% glycerol, 1 mM ribose	
Protein concentration (mg/mL)	9, 6, 3		
Experimental temperature (°C)	4		
Analysis software	Beamline Software [17,18]; ATSAS 3.0.1 [19]		

b) Experimental parameters for WAXS			

Protein	RBP	RBP + 1 mM ribose	RBP + 5 mM ribose

Experimental station	BL-15A2, Photon factory, Japan		
Detector distance (m)	0.5		
Exposure time (s)	10		
Wavelength (Å)	1.2		
Background buffer	50 mM Tris pH 7, 50 mM NaCl, 10% glycerol	50 mM Tris pH 7, 50 mM NaCl, 10% glycerol, 1 mM ribose	50 mM Tris pH 7, 50 mM NaCl, 10% glycerol, 5 mM ribose
Protein concentration (mg/mL)	16	20	17
Experimental temperature (°C)	RT	RT	RT
Analysis software	SAngler [21]

**Table 2:** SAXS data analysis. SAXS data was analysed using ATSAS version 3.0.1. The table provided here shows the values of Guinier R_g_, real space R_g_, D_max_ and qR_g_ range used for Guinier analysis for the RBP SAXS datasets.

	RBP (9 mg/mL)	RBP (6 mg/mL)	RBP (3 mg/mL)	RBP+ribose (9 mg/mL)	RBP+ ribose (6 mg/mL)	RBP+ribose (3 mg/mL)
Guinier R_g_ (nm)	2.1	2.1	2.1	2.0	2.0	2.1
Real space R_g_ (nm)	2.1	2.1	2.2	2.1	2.1	2.1
D_max_ (nm)	8.6	8.4	8.3	8.3	7.6	7.4
qRg range	0.4–1.2	0.4–1.2	0.4–1.2	0.3–1.2	0.3–1.3	0.3–1.3

## 3 Experimental Design, Materials and Methods

### 3.1 Sample preparation

Commercially synthesized and cloned rbsB gene in pET-15b vector (GenScript) was used for expression in *E. coli* BL21(DE3) cells. After cell lysis by sonication, and centrifugation, supernatant was used for two step purification using Ni-affinity column (IMAC Sepharose, GE healthcare) and size exclusion column chromatography (16/60 Superdex 75, GE healthcare). Purified protein in buffer A (50 mM Tris pH 7.0, 50 mM NaCl and 10% (v/v) glycerol) was further concentrated. Protein concentrations were determined using Pierce™ BCA Protein Assay Kit (Thermo Scientific) using the manufacturers protocol. The samples, and background buffers from the size-exclusion column runs, were frozen in liquid nitrogen and shipped to the synchrotron beamlines for data collection. For RBP-ribose sample preparation, the protein sample and background buffer were spiked with d-ribose.

### 3.2 Data collection and analysis

SAXS data was collected at beamline BM29, European Synchrotron Research Facility, Grenoble, France **(**ESRF) [16]. Concentration series experiment was performed at three different protein concentration (3 mg/mL, 6 mg/mL and 9 mg/mL) for both unliganded and liganded RBP so that the concentration dependence can be assessed. To minimize radiation damage, protein samples were continuously moved during X-ray exposure. Azimuthal averaging, frame averaging (up to 10 frames) and buffer subtraction were performed for each dataset using the beamline software [17,18], SAXS datasets were further analysed using ATSAS version 3.0.1 [19].

Samples for WAXS experiment were prepared in the same way as described above. WAXS data was collected at beamline BL-15A2, Photon Factory, Tsukuba, Japan [20]. Since WAXS signal is weak, up to 60 frames were collected and combined in SAngler [21].

All dataset files (accessible from SASBDB [1], see Specifications Table) contain three columns containing momentum transfer (q), Intensity (I) and error associated with intensity. Data collection parameters are provided in Table 1. Differences observed in size parameters upon ribose binding to RBP are provided in Table 3.

**Table 3:** Comparison of sizes obtained from SAXS data of RBP in presence and absence of ribose. The values of R_g_ and D_max_ observed at three different concentrations were averaged and shown here.

	RBP	RBP + ribose
Average R_g_ (nm)	2.10 ± 0.01	2.05 ± 0.02
Average D_max_ (nm)	8.43 ± 0.15	7.76 ± 0.47

## Limitations

None.

## Ethics Statement

All authors have read and follow the ethical requirements for publication in Data in Brief and confirm that the current work does not involve human subjects, animal experiments, or any data collected from social media platforms.

## CRediT authorship contribution statement

**Jagrity Choudhury:** Validation, Formal analysis, Investigation, Data curation, Writing – original draft, Writing – review & editing, Visualization. **Kento Yonezawa:** Methodology, Validation, Investigation. **Anu Anu:** Investigation. **Nobutaka Shimizu:** Conceptualization, Methodology, Validation, Formal analysis, Investigation, Resources. **Barnali Chaudhuri:** Conceptualization, Methodology, Validation, Formal analysis, Investigation, Resources, Writing – original draft, Writing – review & editing, Project administration, Funding acquisition, Supervision.

## Data Availability

RBP SAXS-WAXS 1 (Original data) (SASBDB https://www.sasbdb.org/).
